# Identifying Individuals at High Risk for Permanent Disability From Depression and Anxiety

**DOI:** 10.3389/fpsyt.2020.00740

**Published:** 2020-07-31

**Authors:** Eva Behrens-Wittenberg, Felix Wedegaertner

**Affiliations:** Department of Psychiatry, Social Psychiatry and Psychotherapy, Hannover Medical School, Hannover, Germany

**Keywords:** absenteeism, disability, depression, anxiety, mental health, return to work

## Abstract

Mental illnesses, among which depression and anxiety are most common, are the leading cause for permanent disability. It is of interest to know what case characteristics determine if a person returns to the work force in spite of mental illness to tailor specific interventions. So far, there has been little research into this field. In the present study a sample of 202 workers who had permanent disability due to depression/anxiety were followed-up on. 22% started some kind of work during follow-up in a period of 30 to 75 months. Logistic regression analyses showed that quick progression into disability, short period of disability, younger age, adequacy of income replacement through insurance benefits and no previous disability predicted return to work. These findings also allow to identify individuals at high risk for progression into permanent disability that can benefit from supporting interventions.

## Introduction

With 17% mental health issues, primarily depression and anxiety, have a high prevalence within the working population (1). They are associated with long sick leave periods (2). Affective disorders are the most common (1).

One of the major consequences of mental disorders is the loss of one’s ability to work and hence the loss of the various positive aspects associated with work (3). Work can have a therapeutic effect without actually being therapy. As sense of accomplishment and effectiveness at work rise, feelings of overwhelming exhaustion and cynicism, which are typical aspects of depression, decline (4). Good work mobilizes, provides a daily routine and has a stabilizing effect (5). In contrast, work that is experienced as straining and psychologically demanding while at the same time as uncontrollable and lacking social support is predictive for depression (6–8). The experiences an individual makes are highly dependent on biological disposition and psychological constitution, which determine individual coping strategies (9). Work also provides financial security, which protects against social decline (10). It enables participation in society and provides a sense of purpose and identity (11). Accordingly, people with mental issues who are not in the workforce lack a major aspect of recovery, namely “good” work. Even those employees with mental issues that cannot show their full potential benefit from working or returning to work rather than not doing so (12–14). Not surprisingly, the association between unemployment and its negative health consequences has been confirmed by numerous studies (15).

It is in the general interest to prevent long-term sick leave from work or retirement as this causes costs for everyone (16, 17). This is even more important for mental disorders as their occurrence earlier in the life-span increases the associated costs (18). Programs that promote reactivation are well investigated and mainly include therapeutic interventions focused on work-related strategies (11, 19–21). The evidence for subject-related variables, however, is heterogeneous (2). It is assumed that younger age and higher premorbid income are beneficial (22). Eventual permanent disability is more likely if 42 or more sick days are accumulated (23). There seems to be no evidence for an effect of occupational status and educational level on reactivation (22). Findings concerning socioeconomic status and reactivation are inconsistent (24).

Still some individuals affected by mental disorder return to work quickly despite of great adversity whereas others retire permanently. A systematic analysis of why these people reactivate has not been made to date. This is most likely due to a very limited sample of those who un-retire (25). The reactivation rate from statutory occupational disability pensioning has been estimated at less than 6% (26).

The present study identifies factors that influence permanence of occupational disability for the first time. The individual characteristics of those who returned to work and those who do not are reported.

## Method

### Design

**Figure 1:**
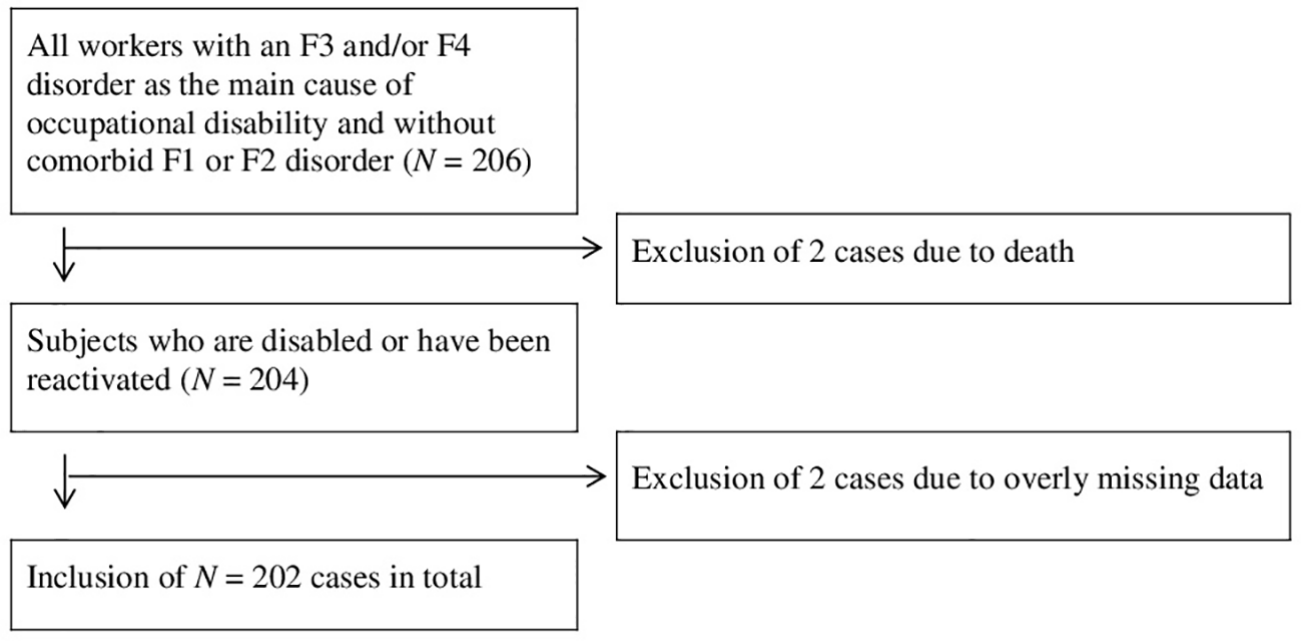
Sampling strategy.

The prospective cohort study was done with data from a German insurance company. Sample size was determined by power analysis for an effect size of 1.3 at *β* = 0.800 and *α* = 0.050. Data was sampled in spring 2018. Included were cases that held a private occupational disability insurance and had occupational disability due to an affective (ICD-10: F3, 73%) or neurotic disorder (ICD-10: F4, 27%). Exclusion criteria were comorbid substance use disorders (ICD-10: F1) and/or schizophrenic disorders (ICD-10: F2). The morbidity was extracted from the subjects’ files which included medical reports, self-disclosures, written correspondence and insurance documents. Cases were sampled from May 2018 backwards by date at application for disability benefits until the required sample size was slightly overreached as determined by power analysis (*N* = 206). From these 4 were excluded because of missing or implausible data. The flow chart (**Figure 1**) illustrates the sampling procedure.

### Variables

The following variables were examined.

Disability at the time of data collection versus return to work.

Sick days: days from start of absenteeism from work until either the date of reactivation or end of observation period.

Income replacement rate: the proportion of insurance benefits to premorbid net income.

Age at onset of disability: age at recognition of disablement was determined from medical statements from the files.

The insurance value is the discounted sum of future pension payments. It was obtained from the insurance documents.

Socioeconomic status (SES): SES was determined with the International Socio-Economic Index of occupational status [ISEI-08, (27)]. The ISEI-08 scale ranges from 13 = *worker in agriculture and animal husbandry* to 78 = *doctor, university professor*. 7 cases were excluded from the analysis due to missing values.

Sick days until disability were calculated from start of mental illness until recognition of disablement. This date was naturally *before* the day of application for benefits.

If a period of previous disablement was present could be determined from the records.

Residency was obtained through the zip codes of the postal address. We differentiated between the states of the former East and West Germany. Post codes with first digits 39, 1-19, 98 and 99 were classified as the former, the remaining codes as the latter.

Unterminated employment was obtained from the records. Subjects had to report when applying for disability benefits if their place of work was still available to them.

The information on the main diagnosis for occupational disability was extracted from the subjects’ medical reports.

### Statistical Analyses

Statistical advisory and verification of statistical procedures were conducted by the Institute for Biometrics of the Hannover Medical School. Data was analyzed using SPSS^®^ 25 (IBM Corporation, Armonk NY, USA) for Windows^®^. Single missing values were assumed to be missing at random.

The sample was split by disability (yes/no) at the time of data acquisition. Examined variables were split at the median if the values were interval scaled to obtain binary variables for logistic regression analyses. Variables with *p*-values ≤ 0.200 were included into multivariate logistic regression modeling as recommended by Hosmer, Lemeshow und Sturdivant (28). The least significant variables were eliminated using stepwise backward selection. Only statistically significant design variables with *p* ≤ 0.050 were added to the final multivariable binary logistic regression model.

### Ethics Committee Approval

The study was approved by the Ethics Committee of the Hannover Medical School (approval number: 3679-2017).

## Results

### Sample Characteristics

51% of the subjects were female. Age ranged from 24 to 61 years with a mean age of 43 years (*SD* = 8). 60% had completed 10 years of schooling while 31% had 12 or more years of schooling (9% indicated to have other schooling). 80% had some form of additional vocational training. All but two subjects were German citizens (99%). Subjects were located representatively in East (23%) and West (77%) Germany (29).

73% of the sample was disabled predominantly due to an affective disorder (F3) while the rest had other disorders of the neurotic spectrum. At the time of data acquisition 77% of subjects were still disabled, 22% un-retired during the period of observation. The mean period of disability was *M* = 1.393 days (*SD* = 530, *Med* = 1501) or 46 months. This means that each subject had received around 4 years of disability benefits by the end of observation. On average, 1.575 days (*SD* = 1.709, *Med* = 974 days), or 52 months, passed between onset of psychiatric problems and recognition of disability. Subjects’ average and median age at the time of disablement was 39 years (*SD* = 8, *Min* = 18, *Max* = 56). In total, 41 subjects (20%) had one or more previous periods of surmounted disability. Subjects’ average SES was 46 ISEI-08 status points (*SD* = 13). The sample was split at the median SES (*Med* = 45) into two groups with *low* and *high* SES. Premorbid net income was 35.861 Euro/a (*SD* = 20.712). The average insurance value was 135.815 Euro (*SD* = 84.754). The median income replacement rate was 31%, i.e., half of the subjects received only 11.117 Euro of insurance benefits or less. At the time of data collection 54% of the subjects indicated that their old job was still available. See **Table 1** for the descriptive statistics.

**Table 1:** Descriptive statistics.

	*Mean*	*SD*	*Min*	*Max*
Age (years)	43	8	24	61
Sick days	1.393	530	59	2.125
Sick days until disability	1.575	1.709	212	12.327
SES (SES status points)	46	13	19	70
Premorbid net income (Euro)	35.861	20.712	5.056	119.647
Insurance value (Euro)	135.815	84.754	13.694	379.995
Income replacement rate (%)	31	21	6	130
Gender (% female)	51			
Education (%)				
10 years	60			
12+ years	31			
Other	9			
Vocational training (% yes)	80			
Citizenship (% German)	99			
Residency (% West Germany)	77			
Reason of disablement (% F3)	73			
Disability status (% return to work)	22			
Previous disablement (% yes)	20			
Unterminated employment (% yes)	54			

SD, standard deviation; SES, Socioeconomic status; F3 affective disorder.

### Logistic Regression

The results of the univariate logistic regression analysis are shown in **Table 2**. 8 of 12 variables had an isolated significant effect on disability with *p* ≤ 0.200: Duration of disability, income replacement rate, sick days until disability, age at onset of disability, insurance value, previous occupational disability, place of residence, and unterminated employment.

**Table 2:** Results of univariate logistic regression analysis.

Univariate logistic regression analysis: Influence of subject characteristics on disability (working vs. disabled)			
	*p-value*	*OR*	*95% CI*
*Dichotomous variables*			
Less than 1.501 sick days during current period of disability (yes)	0.000*	15.625	5.348–45.455
Income replacement rate greater than 25% (yes)	0.062*	1.926	0.968–3.831
Less than 974 sick days from onset until disability (yes)	0.000*	4.926	2.208–10.989
Age at onset of disability younger than 38 years (yes)	0.035*	2.132	1.054–4.310
Insurance value greater than 147.428 Euro (yes)	0.018*	2.254	1.151–4.417
Socioeconomic status ISEI-08 > 45 (yes)	0.666	1.158	0.596–2.248
Preceding period of occupational disability (no)	0.090*	2.381	0.874–6.494
Residence in East Germany (yes)	0.161*	1.697	0.810–3.546
Unterminated employment (yes)	0.077*	1.882	0.933–3.796
Main diagnosis of occupational disability is affective disorder (yes)	0.452	1.353	0.615–2.976

The dichotomous dependent variable is disability at the time of data collection. *Results with p ≤ 0.200 are significant. Abbreviations: OR, odds ratio; CI, confidence interval.

Multivariate logistic regression analysis revealed the final prognostic model for continuous disability. 4 variables were included in the final model as listed in **Table 3**. Shorter period of disability, faster progression into disability, young age and no preceding disability were all independent factors that promoted rehabilitation from disability. Longer period of disability, slower progression into disability, older age and preceding disability worsened prognosis strongly. The model fit can be considered good (Nagelkerke’s *R²* = 0.418; Hosmer-Lemeshow *χ²* (7) = 5.073, *p* = 0.651).

**Table 3:** Results of multivariate logistic regression analysis.

Multivariate logistic regression analysis: Influence of subject characteristics on disability (working vs. disabled)			
	*p-value*	*OR*	*95% CI*
*Dichotomous Variables*			
Less than 1.501 sick days during current period of disability (yes)	0.000	14.265	4.622–44.028
Less than 974 sick days from onset of illness until disability (yes)	0.000	5.200	2.087–12.955
Age at onset of disability younger than 38 years (yes)	0.020	2.856	1.182–6.897
Preceding period of occupational disability (no)	0.033	3.760	1.115–12.685

The dichotomous dependent variable is pension status at the time of data collection. Abbreviations: OR, odds ratio; CI, confidence interval.

## Discussion

Although the most common mental illnesses of the affective and neurotic spectrum are treatable and many patients can enjoy full recovery some cases take long or chronify on a bad functional level. The present study provides an insight into the impact of individual aspects that promote or impede reactivation after disablement. The present study takes into account to what extent the previous place of work is still available after prolonged absence from work and insurance benefits that replace premorbid income. These are, of course, aspects of a society with strong employees’ rights and social security. A study including these important factors of well-being has not been published, presumably because it is difficult to obtain this data.

Our results show that a fast progression from onset of illness to disablement as well as a short period of disability, a younger age and no preceding period of disability increase the chances of overcoming occupational disability and returning to the workforce. Interestingly, no effects were found for socioeconomic status, suggesting that the societal factors mentioned above may ameliorate the additive detrimental effects of low SES and illness on re-employment. Main cause of occupational disability (F3 and/or F4) had no statistically significant differentiating effect either, which is plausible because the prognosis of loss of function may be regarded as independent from diagnosis. Isolated effects with *p* ≤ 0.200 were found for income replacement rate, insurance value, place of residence and for unterminated employment.

Some of these findings replicate results from literature and some are new.

It was surprising to find differentiating factors in these subjects, who were - on average - ill for more than three years. The finding that a relatively swift move from absence from work to disability enhances the likelihood of reactivation corresponds with notions about chronification of depression (3). It is not surprising to see that the likelihood to return to work decreases with length of disability. It has been shown that the risk of premature retirement highly increases if absence from work exceeds 42 days (23). We show that the risk increases even more with each passing day. Furthermore, the finding that a younger age increases the likelihood of reactivation was replicated (22). Those who were in their first period of disability had a higher chance to overcome it. This finding is in line with previous research (2) and general medical experience. Still, it was surprising to find such a strong effect size. All these results underscore the necessity for return to work interventions that occur: 1. early after onset of psychiatric problems, 2. are targeted especially at younger and middle-aged employees and 3. are sustainable to prevent repeated disablement.

Another finding that partly goes along with previous research concerns socioeconomic and monetary variables. In our sample socioeconomic status had no effect on reactivation. This means that reactivation seems to be no more likely for subjects with higher income than for those with lower income. Interestingly, higher insurance value was associated with better outcome and, to a lesser extent, income replacement rate. It is understandable that high insurance value is confounded by age because it is the product of the remaining contract term and annuity. However, the income replacement rate was surprisingly low. Half of the subjects only received monthly benefits around 250 Euro and those who received more seemed to fare better with regard to reactivation. At first, this seemed counterintuitive. One might think that the smaller the loss of income the smaller one’s incentive to return to work in spite of illness. Instead, the higher reactivation rate of the well-insured suggests that not having to face the double jeopardy of mental illness and financial difficulties may have more capacity to focus on recovery.

These isolated effects merit further investigation. In the multivariate model a lower number of sick days, younger age and no preceding disability were found to be strong predictors for the reversibility of invalidity.

In the different systems of social security therapeutic interventions for those who have left the workforce because of health reasons are rare. There exist, of course, many interventions with the goal of return to work during the period of absenteeism from work (30, 31). However, these interventions target employees before occupational disability formally recognized. The present sample was already disabled and most likely took part in most of these interventions. For these people there simply exist no structured interventions. Probably, this group is generally assumed to have little chances of reactivation, not least because of reported low reactivation rates.

It is quite astonishing that, in the present sample of private occupational disability insurance clients, the reactivation rate was 22%. This is almost four times higher than what is expected in the public disability insurance. The most likely cause for this is that disability is not a permanent state in private occupational disability insurance. All rehabilitative interventions end in public disability insurance when premature retirement is reached. Subjects in private occupational disability generally stay in contact with their insurer. In the public disability insurance the status of being disabled is typically not questioned once reached and clients get transferred into old-age pensioning later on. While retirees are reexamined in both systems at regular intervals one may assume that the rehabilitative impetus is smaller in the public insurance sector.

When interpreting the present findings, some considerations also have to be made with regard to data quality. It is worth mentioning that contracts in private occupational disability insurance may be terminated by settlement. These cases would have been lost to follow-up. In our sample we found no cases that ended in settlement simply because the insurance doesn’t use this practice, which can be used to the disadvantage of the client.

Although the reactivation rate is already very high, it may still be underestimated. Clients are obligated to report, if they resume work, but may not always have done so. This would only become known when the insurer re-evaluates the cases which it does roughly every two years. Therefore, it is possible that the real number of reactivated subjects is higher than the one seen in the data.

Furthermore, the amount of sick days until disability might not depict the onset of mental illness correctly. Sick days were computed based on data obtained from claim forms and medical records. Although this is a relatively reliable source, the number of days under morbidity can safely be assumed to be higher than indicated. Absence from work typically occurs at latency after the onset of psychiatric problems. This makes early interventions seem even more important.

In 2017, 26% of the households in Germany had private occupational disability insurance (32). Although the results of this study strictly speaking only apply to those privately insured, it shows that interventions for people with occupational disability in general have great economic potential. Subjects with depression and/or anxiety retire early and lose many productive years. At the same time the situation of these people is different from those who suffer from the other major causes of disability: musculoskeletal disorders, cerebrovascular diseases and cancer. Their disability is not due to the physical loss of a functionally relevant part and should be reversible.

Therefore, it is of great interest not only for the individual but also for the general public to encourage and support people with mental illness even after retirement. This study did one of the first steps in this field of research. It examined workers who are or were on permanent occupational disability and tried to identify variables that facilitate or impede the return into the workforce. The findings should be used to develop interventions that target middle-aged or younger workers at the beginning of psychiatric impairment.

Future research should examine additional variables that might predict return to work. For example, monetary variables should be investigated even more. The precise interaction between monetary incentive and reactivation is still unclear. At least our results don’t support the opinion that smaller disability pensions incentivize. Instead, financial security seems to be helpful during rehabilitation from psychiatric disablement.

The results should help to identify individuals at high risk for long-term chronification at low functional level at an early stage and tailor specific interventions. The authors currently test such an intervention with the goal of reactivation and placement into a problem-compatible value-generating new job. The intervention targets employees at the beginning of their absence from work. It focuses on the individual fit between personal resources, appropriate treatment and work-related factors with the goal of vocational reintegration and to ensure social participation as a major factor of recovery. If this is able to increase the ratio of those who stay in the work force remains to be seen. First results, though not yet statistically significant, are encouraging.

## Data Availability Statement

The datasets generated for this study are available on request to the corresponding author.

## Ethics Statement

The study was approved by the Ethics Committee of the Hannover Medical School (approval 131 number: 3679-2017). Written informed consent was not required as per local legislation and national guidelines.

## Author Contributions

EB-W collected and analyzed the data and wrote the manuscript. FW designed the study, interpreted the data, and wrote the manuscript. All authors contributed to the article and approved the submitted version.

## Funding

Data acquisition was funded and supported by Debeka Versicherungsverein a. G. The funder Debeka Versicherungsverein a. G. was not involved in the study design, collection, analysis, interpretation of data, the writing of this article or the decision to submit it for publication.

## Conflict of Interest

The authors declare that the research was conducted in the absence of any commercial or financial relationships that could be construed as a potential conflict of interest.
